# Delivery of Multiple Child and Maternal Health Interventions during Supplementary Immunization Campaign in Rwanda, 2013: Lessons Learnt

**Published:** 2018-08-02

**Authors:** Hassan Sibomana, Muhoza Jered, Celse Rugambawa, Jethro M. Chakauya, Messeret E Shibeshi, Joseph Okeibunor, Richard Mihigo, Rajesh Bhaskar

**Affiliations:** 1 Ministry of Health, Rwanda Biomedical Center, Rwanda; 2 MOH Rwanda; 3 World Health Organization Country Office, Rwanda; 4 World Health Organization Inter-Country Support Team, Harare, Zimbabwe; 5 WHO AFRO; 6 WHO Consultant, WHO Rwanda

**Keywords:** Antigens, Campaign, Routine, Survey, Vaccination, Immunization, Vaccines

## Abstract

**Objective:**

This paper assesses and describes the estimated coverage of the Measles Rubella (MR) campaign in each district; the national estimate of coverage for Human Papilloma Virus (HPV) vaccination campaign and Vitamin A supplementation simultaneously implemented in 2013.

**Methods:**

We applied descriptive statistics and epidemiological tools to the outcomes of the campaigns to assess the coverage achieved on the different child and maternal health interventions. We also assessed the Adverse Events following Immunization (AEFI) where the evaluation was used at the same time to assess the routine immunization performance coverage for children 12-24 months for all childhood antigens, Tetanus Toxoid coverage among mothers of infants, combined with routine immunization performance evaluation, skilled delivery and bed nets use in Rwanda.

**Results:**

Results indicated that among the eligible targets, 97.5% received MR vaccine, 91% received HPV doses, and 83% got Vitamin A. The integrated vaccination of MR with HPV did not result in any serious AEFI. Coverage for antigens and doses given early in life was above 95% with card retention of 80%. BCG to measles dropout by card was 8.5%. Main reasons for non-vaccination indicated need for more specific immunization education. About 96.8% of mothers delivered in health institutions and 95% of the mothers slept under bed nets the night before the survey.

**Conclusion:**

Rwanda successfully implemented an integrated coverage evaluation survey of the integrated vaccination campaign and routine immunization with statistically valid estimates. We drew lessons that information on routine immunization can be collected during post campaign survey evaluations. The district estimates should guide the programme performance improvement.

## Introduction

Immunization programmes in the African Region have recorded remarkable successes in the last four decades, contributing to the eradication of smallpox, elimination of polio, control of measles and reduction by 99% of the incidence of other vaccine-preventable diseases^1^. Similarly, tremendous progress has been made in the development of new vaccines, along with increasing access to new and underused vaccines in the lowest income countries.

The Rwanda the Ministry of Health provides routine vaccination coordinated through the national expanded programme on immunization. Between 1978 and April 2013 a total of 12 antigens had been introduced into the nation’s immunization system. These include antigens such as Bacillus Calmettie Guuérin, Diphtheria, Pertussis, Tetanus, Oral Polio Vaccine, Measles, Hepatitis B, Haemophilus influenza type b, Pneumococcal Vaccine, Human Papilloma Virus, Rotavirus and Measles-Rubella antigens to prevent and control vaccine-preventable diseases. The impact of vaccination is reflected in the absence of any indigenous wild polio virus since 1993, achievement of the measles pre-elimination target and sustained maternal neonatal tetanus elimination since 2014. There has also been a consistent reduction in infant and child mortality in the country.

The programme has since developed a Measles Elimination Strategic Plan 2012–2020 in line with the WHO African Region technical guidance that aims to eliminate congenital rubella syndrome and introduce rubella-containing vaccine using the Measles Rubella (MR) formulation. It targets children 9 months -14 years to reduce the susceptible pool to be followed by introduction of MR vaccine into the routine immunization schedule. The introduction of the second dose of measles vaccine follows at 18 months of age with aim of achieving high performance coverage to prevent accumulation of susceptible and reach goal of elimination.

Advancing to reduce cancer of the cervix, Rwanda was among the first countries in the region to introduce the vaccine against Human Papilloma Virus (HPV) in 2011. At the time of the survey, a total of 2 cohorts of the target age group of school girls of 9-13 years had received three doses each giving to a total of six rounds of vaccination. During this campaign, taking advantage of the country’s high school enrollment rates the vaccine was integrated with MR but delivered through schools targeting 12-15-year-old girls.

WHO recommends to Member States to conduct periodic assessments of immunization coverage using its standard survey guidelines to validate the reported coverage. Assessing coverage through high-quality surveys is an essential component of monitoring progress towards elimination, eradication or control of vaccine-preventable diseases^2^. Various methods to validate performance coverage including cluster surveys are routinely used in the immunization program^3^ and to assess reasons for non-vaccination and the success of communications strategies^2^. Rwanda thus conducted an evaluation of the integrated MR, HPV and Vitamin A supplementation campaign interventions through a coverage survey validation that was endorsed by the Inter-Agency Coordination Committee (ICC) to document lessons. The outcomes of the evaluation are presented in this paper.

## Methods

The evaluation adopted the household population-based stratified two-stage cluster survey design. The study population consisted of children, who were eligible to receive MR (9–168 months) and Vitamin A (6–59 months) during the SIA. It also included girls aged 9-13 years eligible for previous doses and girls aged 12-15 years eligible for current dose of HPV. Children aged 12-23 months were evaluated of full routine immunization coverage, and mothers of infants aged 0–11 months for routine immunization with TT. Assessment data were collected in 2013, immediately after the campaign.

A total of 8563 Children aged 12-23 months; 11,941 women of reproductive age and 1366 Female adolescents were included in the sample. The WHO cluster sampling technique was used to select participants. Thirty (30) clusters in each of 30 districts were selected following probability proportional to size (PPS) method. Local enumeration areas were used to select clusters for the evaluation. The list of sectors used was provided by Central Statistics Office (CSO) to all teams with a list of clusters and enumeration areas surrounding clusters. Ten children were selected in each cluster by simple random sampling method. Nine hundred clusters were selected in the country targeting interviewing of 900 respondent mothers or caregivers.

The study aimed to get both national and district level estimates through the survey using immunization coverage standard questionnaire adapted from WHO Cluster Survey manual for all interventions, where applicable. Assessment of all the interventions was done at district level for both campaign and routine antigens with the exception of HPV where coverage was estimated nationally. The immunization coverage survey questionnaire captured respondents’ socio-demographic information for target age groups, measles-rubella (age 9-168 months) and vitamin A (age 9-59 months), HPV received during SIA (girls 12-15 years) for previous doses (girls 9-14 years), routine vaccinations during the first 2 years of life (fully vaccinated defined as having received all antigens), Tetanus Toxoid (TT) vaccinations for women of childbearing age, place of delivery, use of bednets among infants and mothers the previous night from the survey, reasons for not receiving vaccinations, sources of information about SIA and SIA messages received.

Oral informed consent was obtained from parents or caretakers. The survey protocol was approved by the Scientific and Ethics Committee of the Ministry of Health of Rwanda.

All the survey teams were trained for 2 days that included practical field exercises and were supervised in the field. Questionnaires were checked and immediate feedback provided by phone calls. The 3 page-questionnaire took an estimated 15 minutes to complete for SIA data only and additional 10 minutes each for the routine child and TT sections. Immediately after field work ended, all teams were gathered for a half-day debriefing about experiences during field work and lessons learned.

### Statistical analysis

All analyses were done in Epi Info using the sample survey procedures. The sampling structure took account of stratification, sample weighting for national estimates, and clustering. The calculation of the SIA and routine coverage rates was done using descriptive statistics. We also analyzed time between birth and routine vaccinations and the interval between doses with immunization cards to assess adherence to WHO recommended time intervals^4^,^5^.

## Results

### Socio-demographic information

Data were available on 8436 of the expected 8704 children for MR vaccine; 3939 of 4100 of children for vitamin A; 1366/1621 for HPV (girl child 12-14 years old); 8563/8773 for children 12-23 months for RI assessment 11,941/12,399 mothers of infants 0-11 months for TT. Eighty five percent of the respondents were from rural while 12% were from rural areas. The age group distribution for mothers indicated that 28% were below 30 years; 42% were between 30-40 years, and 30% were above 40 years. About 80% of the mothers had either primary or secondary education while only 20% were with no formal education. Agriculture was the main business of mothers and specifically 52% were farmers while 13% daily laborers. Christianity is the dominant religion, Catholics, Protestants and Adventists together formed >90% of respondents. Less than half (40%) women had 1-2 children while 22% had >3 children, 32% had one child.

### Integrated Vaccination Campaign activities

Overall the coverage was 97.5% for the MR vaccine, 83% for Vitamin A; 91% for HPV dose achieved during integrated SIAs. Eighty-seven percent of the 8436 respondents had an immunization card. The crude coverage of MR vaccination was 97.5% as compared to 102% reported by the programme while the national coverage by card was only 84%.

Card retention rate for Vitamin A for children aged 9 to59 months was 88% with coverage estimate of 83% while coverage validated by card alone was 75%. The reported administrative coverage for Vitamin A (6-59months) was 86%.

### Human Papillomavirus (HPV)

The national HPV card retention rate was 87%. The crude coverage of HPV during the campaign was 91% as compared to 99% reported administrative coverage. The non-response rates for HPV coverage started increasing sharply from HPV1 to HPV3. Ninety percent of girls receiving HPV were in school. The administrative coverage of in-school girls was 98.1% while for out of school was 1.1% indicative of high school enrollment in Rwanda among those surveyed. About half (53%) of the respondents got their vaccination at school and 32% at health centers.

### Source of information on the SIAs

Among the 8266 respondents, only 0.4% reported that they were not informed of the campaign before the implementation. The commonest sources of information on the campaign were radio and Community Health Workers (27% each), followed by community leaders (25%); school children in the family (13%); and health workers in health centers (8%).

### Childhood immunization

The records showed immunization card retention rate was 80%. The coverage for BCG was 99%, and all antigens achieved more than 95% coverage. The rate for full immunization of children from card and recall combined was 94%.

The reasons for non-immunization of children included lack of information and motivation among caretakers. Specifically, 18% indicated that they were unaware of the time of vaccination, while 11% reported that they were not aware of the need for vaccination.

### Mothers of infants 0-11 months

Eighty-seven percent received at least one injection of TT during the latest pregnancy. The proportion of women receiving two or more injections of TT, (TT2+) by card plus recall was 91.7% while only 18% from card only due to poor card retention (Table 1).

The protection at birth from tetanus in last pregnancy depending upon the number of TT injections received was 76% from mothers’ recall and card together. The Government health centers were the major source of providing TT vaccination (97.1%).

Among 11,941 mothers of infants assessed, the card retention rates were only 36%. The vaccination card retention rates were lower among new mothers despite the short time span of one year between child delivery and the evaluation survey.

### Reasons for failure for immunization among mothers

There was a total of 990 inadequately vaccinated mothers where the main reasons cited included being unaware of the need for TT vaccination (25%), and unaware of the need to return for another dose (35%). Other reasons included postponed to another time (20%), lack of faith in immunization (10%), time for vaccination not known (5%) and being unaware of the place of immunization (5%). Of the obstacles mentioned as a reason for not being vaccinated among the 990 respondents who were unvaccinated against TT were as follows: mother was too busy 31%; family problems 23%; child illness 15%; TT vaccine not available 15%; location of vaccination too far 8%; and religious reasons 8%.

### Antenatal care and deliveries

The majority (87%) of the women attended the health center for antenatal checkup at least once during the pregnancy. More than ninety-six percent (96.8%) of the mothers delivered in health institutions. Thus, 97% of deliveries were conducted by skilled persons.

### Bed nets availability and use

A high proportion of mothers (89.5%) had bed nets and 95% of them slept under the nets the night before the survey. The bed-net distribution assessment indicated that 83.8% respondents received bed nets from the health centre during one of their visits for health services while 5.7% bought the bed nets from the market.

## Discussion

Rwanda has been able to achieve conclusively its ambitious target of conducting a nationwide integrated post measles rubella and routine immunization coverage evaluation survey with district-wide statistically valid estimates. The country immunization programme has ably introduced MR vaccine into the system; the HPV vaccine has optimum coverage in the immunization programme as well as through integration with MR campaign.

The immunization card retention rates of 87% for MR campaign services allowed reliable estimation of the performance. The result confirmed that the target of coverage rate of >95% for MR vaccination was achieved by crude coverage of 98% and 84% validated by card with confidence intervals between 1% and 3% indicating a high precision of estimates for both. For 96% of those who received MR dose during the campaign, it was their second opportunity to receive the measles vaccine indicating the accessibility of the services through routine immunization programme.

The national estimated coverage for HPV was 91% and by card was 87%. The vitamin A coverage for the country was 85% while the reported coverage for Vitamin A was 86%. The optimum card retention for childhood immunization records documented at 80% may indicate awareness or value given by caretakers in keeping health records of their children. The routine immunization coverage for BCG was 99% and all the antigens achieved more than 95% coverage. The fully immunized child from card and recall was 94% that is above the minimum target of 90%.

The dropout rate for BCG to measles by card and recall was 3.1% while by card only it was 8.5% which indicates fairly good service utilization. The reasons cut across all antigens and have implication for activities to sensitize and educate the people not only on the benefits of vaccination but also where and when they can get the vaccination. The role of such deliberately planned and implemented enlightenment activities for successful immunization was articulated in studies on vaccine refusal in Nigeria^6^–^8^.

The vaccination cards were also deficiently completed thus making monitoring of vaccination statuses difficult. It is evident that almost all the antigens registered a coverage rate of >95% by card and recall together, however, all had lower coverage by card indicating a need to improve record keeping. The doses of routine immunization though given were not recorded on immunization card despite 80% children had cards at the time of the survey. This is a common challenge to tracking vaccination by conventional means^9^,^10^. Measles coverage by card and recall was 97% and 71% by card only. Valid dose for measles was 75%. Almost all (94%) children were found fully immunized with 11 basic antigens from card and history and only 78% fully immunized by card. It is notable that 73% of the children were fully immunized by the age of 1 year (from the card). Compared with administrative coverage of the country from Joint Reporting form and EPI coverage evaluation survey of 2007, the evaluation survey 2013 shows a clear and significant improvement in coverage of all indicators since 2007 and not too much deviation from administrative coverage.

The ANC coverage is fairly good with high health facility delivery. The card retention among young children (12-23mths) was 80%. There is high coverage of bed net distribution and use for both mothers and children in Rwanda.

The TT profile revealed TT2+ coverage among pregnant women was 92%. Protection at birth (PAB) was 76%, while 98% deliveries took place either at health centre or the hospital and attended by skilled person. More than eighty-three percent (83.4%) of pregnant women received bednets during their ANC visits. The results revealed a high utilization of health facilities both for delivery and antenatal care services. These results reflect a success story on the immunization programme in Rwanda.

Despite the success of the assessment, the exercise had a limitation. First is that the campaigns were not designed as studies so no robust analytical approach was built in from the planning stage. However, this did not affect the data as the reporting formats had provided publishable data in the past. The data were carefully cleaned and analyzed to provide necessary information used here. It also provided lessons upon which recommendations are made to improve on future activities. There is need to improve on record keeping. To do this, data collected during the campaigns should be transferred to the immunization cards to avoid failure in human memory.

## Conclusions

Rwanda was able to implement an integrated coverage evaluation survey for the integrated MR, HPV vaccines inlcuding routine immunization surey. The evaluation provided valid estiments for both the campaign and routine immunization with district-wide statistically valid estimates.

In addition, the survey documented that no serious AEFI were reported by integrating MR with HPV vaccination during the campaign. The additional evaluation of bed net use and skilled delivery has demonstrated the successful integrated child and maternal health care delivery systems that exist in the health system in Rwanda.

The participation of district teams in data entry and data cleaning has extensively built the capacity of district teams. They should continue the practice of these skills for periodic assessment of program performance in their respective areas. The lesson from Rwanda should be extensively used for such elaborate coverage evaluation surveys.

## Figures and Tables

**Table 1:** Results of 30 cluster RI evaluation survey-Rwanda 2013

Total Respondents	N= 8563								
	Coverage by Card plus Recall				Coverage by Card Only				Admin Coverage*
Indicators	%	CI: Lower	CI: Upper	DEFF	%	CI: Lower	CI: Upper	DEFF	
Immunization cards available	80%	79%	82%	1.807					
BCG Scar present	98%	98%	99%	1.197					
BCG	99%	99%	99%	1.145	76%	75%	78%	1.646	99%
OPV0	98%	98%	99%	1.352	75%	73%	76%	1.779	
OPV1	98%	98%	99%	1.352	76%	74%	77%	1.784	
OPV3	98%	95%	100%	8.91	74%	72%	76%	1.759	101%
DTPHibHepB1	99%	99%	99%	1.373	75%	74%	77%	1.765	102%
DTPHibHepB3	98%	98%	98%	1.463	74%	72%	75%	1.755	101%
PCV1	98%	98%	99%	1.481	75%	73%	76%	1.76	102%
PCV3	98%	95%	100%	8.91	74%	72%	75%	1.755	101%
Measles	97%	96%	97%	1.435	71%	67%	75%	3.754	102%
Fully Immunized	94%	93%	94%	1.511	63%	61%	64%	1.6568	
Fully Immunized by 1 year	59%	57%	60%	1.587					
OPV3 Valid doses	76%	74%	77%	1.227					
DTPHibHepB3 Valid Doses	69%	68%	71%	1.224					
PVC3 Valid Doses	74%	73%	75%	1.252					
Measles Valid Doses	57%	55%	58%	1.323					

* Joint Reporting Form (JRF) 2012

## References

[1] Larson HJ, Cooper LZ, Eskola J (2011). Addressing the vaccine confidence gap. Lancet.

[2] World Health Organization (2012). Global measles and rubella strategic plan: 2012-2020.

[3] World Health Organization. Africa Regional Office (2006). Measles SIAs Evaluation Guidelines.

[4] Clark A, Sanderson C (2009). Timing of children’s vaccinations in 45 low-income and middle-income countries: an analysis of survey data. Lancet.

[5] World Health Organization (2009). Measles vaccines: WHO position paper. Weekly epidemiological record No. 35.

[6] Murele B, Vaz R, Gasasira A (2014). Vaccine perception among acceptors and non-acceptors in Sokoto State, Nigeria. Vaccine.

[7] Jegede AS (2007). What led to the Nigerian boycott of the polio vaccination campaign. PLOS Medicine.

[8] Renne E (2006). Perspectives on polio and immunization in Northern Nigeria. Soc Sci Med.

[9] Sanou A, Simboro S, Kouyaté B (2009). Assessment of factors associated with complete immunization coverage in children aged 12-23 months: a cross-sectional study in Nouna district, Burkina Faso. BMC International Health and Human Rights.

[10] De Wals P, De serres G, Niyonsenga T (2001). Effectiveness of a mass immunization campaign against serogroup C meningococcal disease in Quebec. JAMA.

